# Asbestos Poverty as a New Paradigm for Multidimensional Urban Sustainability

**DOI:** 10.1007/s11524-026-01063-5

**Published:** 2026-03-06

**Authors:** Gergely Zoltán Macher, Dalma Bódizs, Dóra Sipos, Dalma Schmeller

**Affiliations:** Department of Applied Sustainability, Albert Kázmér Mosonmagyaróvár Faculty of Agricultural and Food Sciences, Széchenyi István University, Győr, 9026 Hungary

**Keywords:** Asbestos poverty, Social sustainability, Social impact, Asbestos regulation, Economic challenges

## Abstract

The popularity of asbestos-containing products stemmed from their fire resistance, thermal insulation properties, and mechanical strength. However, their well-documented adverse health effects led to the prohibition of their use in many countries. This research aims to conduct a comprehensive examination of the often-overlooked social dimensions associated with asbestos, with a specific focus on the affected population’s circumstances and the potential solutions accessible to them. Its analysis encompasses legal regulations concerning asbestos, societal awareness, and the economic implications of asbestos removal from the perspective of those impacted. The findings highlight that the remediation of asbestos-containing products is often contingent on the financial and social conditions of the affected population, posing significant challenges for the economic sector and environmental protection efforts. This research contributes to the development of integrated approaches that address social, economic, and environmental dimensions in tandem. Its originality lies in situating the concepts of social sustainability and socially oriented environmental development within the context of asbestos-related policies. The findings suggest that achieving asbestos-free environments is feasible only through the integration of social dimensions, taking into account the economic and social conditions of the affected communities.

## Introduction

Asbestos poverty (abbreviation: AP) represents a complex and multidimensional issue, with its conceptual underpinnings and assessment approaches still undergoing development. The environmental aspects of this socioeconomic issue have long been acknowledged [52, 79], but the social and economic dimensions have more recently garnered the attention of researchers and experts [12]. Empirically observed changes in the phenomenon, coupled with emerging economic and political challenges, have contributed not only to its enduring presence but also to a more profound understanding of its underlying nature. The fundamental interpretation of AP encompasses the limitations in accessing asbestos removal and substitution services due to financial constraints, rendering such services inadequate, inaccessible, or unfeasible [36]. This challenge manifests in two key problem domains: limited local accessibility, which presents obstacles in both developed and developing nations, and affordability, which remains a crucial focus of investigation in advanced economies [2, 5].

The expanding field of AP research has given rise to new insights, encompassing access to alternative and secure construction materials, the effectiveness and thoroughness of asbestos removal processes, and the mitigation of health risks [19, 60, 77]. At its core, AP represents the inability to satisfy fundamental human needs, such as access to a safe and financially accessible living space [49]. This issue extends beyond the fulfilment of basic, day-to-day necessities, such as ensuring a healthy residential environment, and also encompasses higher-level, socially and culturally determined requirements [67]. The highly diverse approaches introduced by researchers necessitate that AP be understood not only in an objective, environmental, or material context but also by considering its subjective dimensions, particularly those related to the so-called social individual. This involves analyzing the factors contributing to poverty related to asbestos exposure and the impacts of inaccessible services [29]. The AP poses substantial socioeconomic and strategic challenges, yet relatively few institutions have taken action to mitigate its impact [15, 19, 51]. On a global level, the United Nations’ 2030 Sustainable Development Goals (abbreviation: SDGs) prioritize the creation of safe and sustainable built environments under Goal 11 [6, 59].

However, the indicators do not specifically address the assessment of asbestos exposure levels. While European Union (abbreviation: EU-27) countries face analogous dilemmas, partly due to their historical contexts, each economy pursues its asbestos-free objectives under unique national circumstances [55], leading to variations in the scope and nature of AP across countries. This prompts the question of whether policies to mitigate asbestos exposure should be standardized across the EU-27 or whether country-specific, customized approaches would be more suitable. The response chiefly hinges on the dynamics of asbestos exposure and AP in diverse economies. If the phenomenon exhibits convergence, joint solutions should be pursued, whereas if divergence is evident, policies must be adapted to address distinct national obstacles [13]. Despite the existing research on construction standards, health risk reduction, and building safety improvements [3, 66], the academic literature currently lacks a comprehensive analysis examining the convergence of AP.

Therefore, the aim of this paper is to identify the current state and dynamics of AP across the Hungarian municipalities, with a particular focus on its convergence and the factors contributing to this process. The added value of this paper lies in the theoretical and empirical structuring of the dynamics of AP, establishing a logical linkage between its causal factors and resulting outcomes. The analysis adopts a comparative approach, investigating the convergence of AP across Hungarian settlements. To provide a thorough assessment of AP as a multifaceted issue, the research employs standardized measurement techniques and examines the validity of the convergence hypothesis:

- H-1: Informational poverty directly contributes to the persistence of infrastructural and financial AP, as a lack of awareness among the population hinders the initiation of asbestos removal efforts and the utilization of available support programmes.
- H-2: Infrastructural AP exacerbates financial AP, as the absence of proper waste management and asbestos removal facilities increases the associated costs, further worsening the situation of affected individuals.
- H-3: Financial AP reinforces both informational and infrastructural poverty, as low-income households not only struggle to afford asbestos abatement but also have limited access to relevant information and infrastructural services.

This paper seeks to address the gap in existing research by analyzing the convergence of AP, providing an empirical foundation for policymaking. Identifying convergence in AP can inform coordinated policy and financial support responses, while recognizing divergence can inform tailored strategies by individual municipalities. Validating this hypothesis is a necessary step for rational policy design within the Hungarian context, rendering this paper of significant diagnostic and policy-relevant value.

## Literature Review and Conceptual Framework of the Research

Asbestos was widely recognized as a popular construction material for decades; however, due to its adverse health effects, its use has been banned in most countries today [25, 45]. Although the presence of asbestos may appear harmless to the general population, it poses a significant public health risk, particularly when roofing materials degrade and release hazardous fibres into the environment [28, 32, 57, 65]. These airborne fibres, once inhaled, can lead to severe respiratory diseases, including asbestosis, lung cancer, and mesothelioma, often with long latency periods, making early detection and prevention particularly challenging [20, 35]. Moreover, asbestos contamination is not only a human health concern but also an environmental issue, as fibres can accumulate in soil and water sources, leading to prolonged ecological damage [17, 42].

Asbestos cement (abbreviation: AC), a composite material made from a mixture of cement and asbestos fibres, was widely used in roofing, siding, and piping due to its durability, fire resistance, and low cost [9, 44]. Its widespread application throughout the twentieth century resulted in a significant number of buildings still containing AC elements, particularly in residential and industrial settings [70, 79]. Despite the ban, asbestos-containing materials (abbreviation: ACM), particularly aged AC roofing, remain widespread in many areas [1, 24]. Over time, weathering and physical deterioration cause these materials to become brittle, increasing the likelihood of fibre release into the environment [73]. This poses a continuous exposure risk, especially in regions where proper maintenance or replacement is financially unfeasible [62]. The process of asbestos removal, however, presents numerous challenges, as it is both slow and costly [48]. Consequently, many households and communities remain exposed to the health risks associated with asbestos [24]. Furthermore, the safe disposal of ACM remains a significant challenge, as inadequate waste management practices can result in secondary exposure risks [19, 68]. An additional concern arises in disadvantaged regions, where access to essential services, including financial resources, is significantly limited [27]. This socioeconomic disparity places affected populations at a considerable disadvantage compared to the broader society [72]. The primary reasons for the continued presence of asbestos materials are the economic burden of removal and a lack of information regarding the associated health risks [24]. Although financial assistance programmes exist, they are often insufficient to cover the full costs of asbestos abatement, particularly in cases where structural reinforcements or complete roof replacements are necessary [46]. As a result, many affected households remain unable to take the necessary steps to eliminate asbestos hazards from their living environment [63].

The concept of AP refers to social groups and regions where individuals, due to financial constraints or inadequate infrastructure, are unable to eliminate ACM from their living environments. Addressing this issue requires comprehensive policy interventions, including subsidized asbestos removal programmes, stricter regulations on asbestos waste disposal [43], and increased public awareness campaigns to ensure that affected populations are adequately informed about the risks and available mitigation strategies [19, 81]. If a precise definition is sought, AP can be understood as the socioeconomic condition in which individuals or communities lack the resources to remove asbestos-containing materials from their living environments. This creates a persistent public health risk, as prolonged exposure to deteriorating asbestos increases the likelihood of serious illnesses like asbestosis, lung cancer, and mesothelioma [56]. The issue is worsened by social and economic inequalities, as those affected often lack the necessary knowledge, access to professional services, or finances to address asbestos-related hazards [74]. As described, asbestos poverty can be categorized into three principal types:

- *Type I (informational poverty)*: Individuals living in informational poverty regarding asbestos are unaware of the associated health hazards and therefore fail to recognize its potential dangers. These affected individuals frequently lack essential knowledge about the severe diseases that can arise from inhaling fibres released by aging or damaged ACMs. Due to this deficit in information, they neglect to take steps to remove asbestos or implement necessary safety precautions, which ultimately poses significant health risks to both them and their surroundings.
- *Type II (infrastructural poverty)*: This category encompasses individuals cognizant of the health perils posed by asbestos yet lack access to resources necessary for its safe elimination. Those impacted may inhabit localities devoid of expert guidance, adequate waste management infrastructure, or official asbestos abatement initiatives. Consequently, they may be compelled to undertake asbestos removal themselves, escalating their exposure risk, or remain in the presence of the hazardous material without viable means to eradicate it.
- *Type III (financial poverty)*: Those facing financial hardship are aware of the dangers posed by asbestos exposure yet lack the financial resources to facilitate its safe removal and replacement. Even when asbestos abatement programmes provide some level of support, such as free demolition and disposal services, many still lack the funds to procure and install new roofing materials. As a result, affected individuals are often compelled to continue residing in asbestos-containing structures or resort to temporary, suboptimal solutions, further exacerbating health risks and perpetuating social inequalities over the long term.

A common factor across all three forms of AP is that the existing deficiencies in information, infrastructure, or financial resources contribute to the continued presence of this hazardous material in the environment [34]. Asbestos fibres, when inhaled, can cause severe diseases, including asbestosis, lung cancer, and the highly aggressive mesothelioma [10]. The risk is particularly high in households where deteriorating building materials or environmental conditions facilitate the airborne release of asbestos fibres [7, 40]. Populations unable to afford asbestos removal remain continuously exposed to these health hazards [58]. Research has shown that malignant mesothelioma has been diagnosed even in individuals who were not occupationally exposed to asbestos but lived in homes with asbestos-cement roofing materials [38]. This underscores the significant public health threat posed by residential asbestos exposure, particularly in socioeconomically disadvantaged communities.

The notion of the relationship between asbestos exposure and poverty can be situated within the frameworks of environmental justice and energy poverty (abbreviation: EP). The former context indicates that asbestos exposure disproportionately impacts marginalized populations [31], which can be characterized by the AP typology detailed previously. Environmental injustice exacerbates social disparities and the precariousness of the impacted communities [11, 18], wherein policymakers hold a pivotal role in ameliorating these issues [47]. Asbestos exposure constitutes a slow-onset, long-term form of environmental injustice [64]. Although the literature extensively explores the environmental and health consequences of asbestos exposure [19, 27], the methodology for quantifying AP remains understudied. In this context, the measurement approaches and indicator frameworks developed for EP could offer a useful starting point for future investigations [37, 76]. There are notable parallels between AP and EP, as both represent structural challenges with substantial implications for quality of life [23]. EP typically refers to households unable to meet their energy needs due to low incomes, high costs, and insufficient building or appliance efficiency [30].

Analogously, AP could describe households where residents struggle to adequately avoid asbestos exposure in their living environment. Just as EP disproportionately affects socially excluded or economically disadvantaged regions, AP may disproportionately burden lower-income households and marginalized communities. Objective measures of EP often focus on household financial circumstances, comparing income levels to the proportion of spending dedicated to energy costs [80]. The objective approach typically relies on household income, which is compared to the proportion of energy expenditure. Indicators such as the 10% threshold, the low-income high costs (abbreviation: LIHC) indicator, minimum income standards, and energy burden [78] are used. Key objective topics for measuring EP include building stock data, heating systems, energy supply options, energy prices, demographic and income data, and policy background [4, 23].

In contrast, the subjective approach is based on self-assessment, focusing on how households perceive their access to energy. Examples include surveys such as the EU-SILC, Eurobarometer, and EQLS in the European Union [39], which assess perceived deprivation, housing conditions, satisfaction, and household expenditures [14]. Similar methods can be applied to measure AP, given that this issue also affects households. Objective indicators could include the age, condition, size, building materials, and asbestos content of buildings, the number or proportion of residents exposed to asbestos, the status of asbestos remediation, and the costs of remediation in relation to household income [33, 41]. The subjective assessment of AP could be based on residents’ personal opinions, such as their level of concern about the presence of asbestos, whether they suffer from any health conditions related to asbestos exposure, and their access to asbestos remediation and available support options [19]. Another important indicator could be the geographical location of the household, as some regions are less developed, which may lead to a higher degree of AP [34].

The concept of AP offers a framework to understand the socioeconomic barriers that hinder effective mitigation of asbestos exposure risks, particularly in marginalized communities. It links environmental justice and EP, acknowledging that disadvantaged groups face challenges such as lack of information, infrastructure, and financial resources. This approach calls for targeted interventions that address both health/environmental and socioeconomic factors. The primary contribution of this work is the introduction of the AP concept, offering a new perspective on asbestos risks in vulnerable communities and demonstrating how structural inequalities sustain these exposures. While much research focuses on the health and environmental impacts of asbestos, few studies explore the socioeconomic factors that prevent at-risk populations from addressing these hazards. Additionally, there is a lack of clear methodologies for assessing AP and understanding its socioeconomic dimensions across various contexts. The key question emerging from this gap is as follows: How can the socioeconomic aspects of asbestos exposure be quantified, and what policy measures are needed to reduce AP and its health risks in socioeconomically disadvantaged communities?

## Methodology

The primary goal of this research is to assess the extent and characteristics of AP in Hungarian municipalities, with a focus on its financial, infrastructural, and informational dimensions. The findings will enhance the understanding of AP and inform the development of targeted policy interventions aimed at mitigating its effects on affected communities.

### Research Design and Approach

This paper employs a mixed-methods approach to examine AP in Hungarian cities and settlements, integrating both quantitative and qualitative methodologies. Given the multidimensional nature of AP, encompassing financial, infrastructural, and informational aspects, the research adopts an interdisciplinary analytical framework. This includes the application of statistical tools and econometric modelling to measure AP, as well as policy content analysis and comparative assessments to evaluate the impact of public policies. The paper aims to identify socioeconomic inequalities, infrastructural deficiencies, and policy interventions, providing insights into their role in shaping AP.

#### Data Collection

The data collection process draws upon multiple reliable sources to ensure a thorough and comprehensive assessment of AP. Authoritative statistical databases, such as Eurostat, the European Environment Agency, and national statistical offices, provide extensive quantitative data on financial poverty indicators, detailed asbestos-related health statistics, and comprehensive information on infrastructure availability. The key indicators examined during the analysis are outlined in Table 1. Additionally, surveys and questionnaires are widely distributed among affected communities to comprehensively evaluate self-reported experiences of AP, detailed perceptions of asbestos-related risks, and extensive awareness of available remediation programmes. Furthermore, a comprehensive analysis of policy and legislative documents, including government reports, European Commission directives, and in-depth asbestos management strategies, is undertaken to compare policy responses.

**Table 1:** Levels of AP and related factors

Indicator	Unit
1 st level AP: Informational deficiency	
Proportion of the population unaware of the health risks of asbestos	%
Proportion of the population unaware that asbestos-cement waste is hazardous waste	%
Proportion of people unaware of free/subsidized asbestos removal programmes	%
Number of informational campaigns within the municipality per year	Units/year
Inclusion of asbestos-related knowledge in educational programmes	Yes/no
2nd level AP: Lack of service accessibility	
Availability of asbestos removal services	km
Availability of official asbestos waste disposal points	km
Number of accredited asbestos removal companies in the county	Units
Number of authorities and experts involved in asbestos removal in the region	Units
Proportion of people with access to at least one asbestos-related service (waste disposal, consultation, decontamination, etc.)	%
3rd level AP: Financial barriers	
Proportion of individuals unable to afford asbestos roof removal	%
Proportion of individuals unable to finance a new roof despite subsidies	%
Proportion of individuals unable to provide the necessary self-contribution for subsidy eligibility	%
Proportion of applicants to subsidized asbestos removal programmes who actually complete the process	%
Amount of asbestos removal subsidies as a proportion of annual household income	%
Policy and regulatory framework	
Existence of a local or regional asbestos removal strategy	Yes/no
Availability of municipal subsidies for asbestos removal	Yes/no
Enforcement of asbestos-related regulations at the local level	Yes/no
Participation/awareness raising	
Number of informational campaigns in the given year	Units/year
Availability of professional consultation at the municipality or other official bodies	Yes/no
Public interest and participation in asbestos removal programmes	%
Number of related civil society organizations and community initiatives in the region	Units

#### Calculation of Poverty Level Values

The quantitative assessment of AP relies on multiple indicators structured across three key dimensions: financial, infrastructural, and informational. Financial indicators include household income relative to asbestos removal costs, availability and accessibility of financial support programmes, and the cost-to-income ratio for asbestos abatement. Infrastructural indicators involve regional availability of asbestos removal and waste disposal services, distance to the nearest certified asbestos removal facility, and the prevalence of ACMs in residential and public buildings. Informational indicators assess awareness levels regarding asbestos-related health risks, public access to asbestos-related information, and participation rates in asbestos awareness campaigns. To construct a composite indicator that captures the multifaceted phenomenon of AP at the local level, we rely on a normalized index system composed of three conceptual dimensions: informational deprivation, service access deprivation, and financial/material deprivation.

Each of these dimensions (also referred to as macro-components) is operationalized through a set of measurable indicators, either continuous (e.g. percentages) or categorical (e.g. binary variables). Let us define *x*_mij_ as the raw, observed value of indicator *j* within macro-component *i* for municipality *m*, where *m* = *1, M, I* = 1, 2, 3, and *j* = 1, …* I*_i_, with *I*_i_, denoting the number of indicators in component *i*. Since indicators may be on difference scales and units, we apply a normalization procedure to render them comparable. Specifically, we use min-max normalization, scaled to a five-point ordinal scale ranging from 1 (very low deprivation) to 5 (very high deprivation). For indicators where a higher raw value reflects greater deprivation (referred to as negative indicators), the normalized score is computed as Eq. (1).

1 $${S}_{\mathrm{mij}}=1+4\times \frac{{x}_{\mathrm{mij}}- {x}_{\mathrm{ij}}^{\mathrm{min}}}{{x}_{\mathrm{ij}}^{\mathrm{max}}- {x}_{\mathrm{ij}}^{\mathrm{min}}}$$

where *x*_ij_^min^ and *x*_ij_^max^ represent the minimum and maximum values of indicator *j* across all municipalities. Conversely, for positive indicators, where higher values are beneficial (e.g. number of local information campaigns), the normalization is reversed as Eq. (2).

2 $${S}_{\mathrm{mij}}=1+4\times \frac{{x}_{\mathrm{ij}}^{\mathrm{max}}- {x}_{\mathrm{mij}}}{{x}_{\mathrm{ij}}^{\mathrm{max}}- {x}_{\mathrm{ij}}^{\mathrm{min}}}$$

This transformation ensures directional consistency across all indicators: higher scores always correspond to worse conditions in terms of AP. Categorical variables, such as binary “yes/no” responses, are first converted to numerical equivalents (e.g. “yes” = 0, “no” = 1) prior to normalization, preserving the intended ordinal meaning. In the absence of domain-specific weights, indicators within each component are assumed to be equally important. Thus, the component score for municipality *m* in macro-component *i* is calculated as the arithmetic mean of its normalized indicator scores (Eq. (3)).

3 $${S}_{\mathrm{mi}}=\frac{1}{{I}_{i}}\sum_{j=1}^{{I}_{i}}{s}_{\mathrm{mij}}$$

If expert-driven or empirically derived weights *w*_ij_ are available, a weighted average may be used in place of equal weighting. To obtain an overall index value for each municipality, we compute a weighted average of the three component scores. Denoting the global weights of each macro-component as *α*_1_,* α*_2_,* α*_3_ (where *α*_1_ + *α*_2_ + *α*_3_ = 1), the final Asbestos Poverty Index (abbreviation: API) is given by Eq. (4). The calculation applied the following weighting factors: *α*_1_ = 0.3, *α*_2_ = 0.3, and *α*_3_ = 0.4. Given the sensitivity of the topic, the municipalities studied are not identified by name but are instead designated as *C-1, C-2, …, C-n* to ensure confidentiality.

4 $${API}_{m}= \sum_{i=1}^{3}{\propto }_{\mathrm{i}}\times {S}_{\mathrm{mi}}$$

This scalar measure enables direct comparison across municipalities, while maintaining interpretability on the 1–5 ordinal scale. The index serves as a synthetic indicator of vulnerability related to asbestos exposure and remediation capacity, informing prioritization in public health interventions and infrastructure support.

## Results

The aggregated data derived from five municipalities reveal stratified patterns of AP, differentiated across three analytically distinct macro-dimensions. These dimensions, informational poverty (1st-degree), service accessibility deprivation (2nd-degree), and material deprivation (3rd-degree), function as overlapping but independent contributors to cumulative environmental vulnerability. The index construction incorporates weighting coefficients (0.3, 0.3, and 0.4, respectively), selected to reflect both the theoretical salience and practical impact of each domain in shaping a community’s ability to manage asbestos exposure risk.

The first degree of AP, represented by informational deprivation, demonstrates the most pronounced inter-municipal heterogeneity. The informational AP captures the extent to which local populations have limited or uneven access to relevant knowledge regarding the presence, risks, and mitigation strategies associated with asbestos. Index scores in this dimension range from a minimum of 1.00 (C-1) to a maximum of 5.00 (C-5), with other municipalities occupying intermediary positions (C-2: 3.87; C-3: 1.80; C-4: 3.18). These findings indicate that even within moderately deprived settings, informational asymmetries can persist independently of material or infrastructural conditions. The data suggest that the provision of information is not necessarily correlated with service availability or economic capacity, pointing toward structural inefficiencies in risk communication frameworks. Informational deprivation therefore emerges as a distinct axis of vulnerability, highly variable, and only partially aligned with broader patterns of poverty, underscoring the critical importance of tailored educational and communicative interventions.

**Fig. 1:**
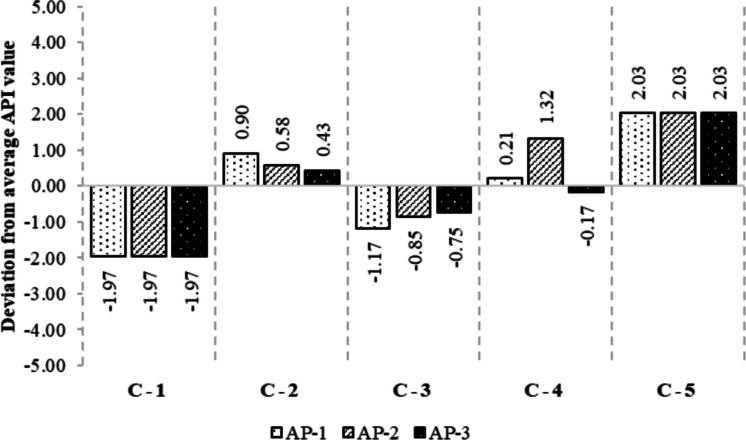
Comparative AP-values across municipalities (C-1 → C-5)

The second dimension, service accessibility deprivation (2nd-degree AP), represents limitations in the availability and reach of asbestos-related public health services, including medical monitoring, remediation assistance, and regulatory enforcement. Figure 1 illustrates the comparative distribution of AP index values across the examined municipalities, providing a visual representation of inter-locality disparities in asbestos-related deprivation. Across municipalities, this domain also exhibits wide variability, with scores ranging from 1.00 (C-1) to 5.00 (C-5). Intermediate values (C-2: 3.55; C-3: 2.12; C-4: 4.29) further highlight significant disparities in infrastructural capacity and administrative responsiveness.

**Fig. 2:**
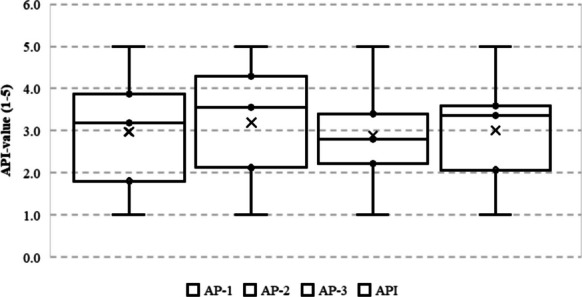
Comparative AP-values across levels (AP-1 → API)

Notably, some municipalities with moderate informational scores suffer from disproportionately high service-related deprivation (e.g. C-4), suggesting a disconnect between knowledge access and the ability to translate that knowledge into action. This asymmetry is particularly salient in contexts where administrative underinvestment, geographical remoteness, or political marginalization impede service delivery. The relative weight of this macro-area in the composite index calculation (0.3) ensures that such infrastructural insufficiencies exert a non-trivial impact on the overall AP profile, amplifying other forms of deprivation and constraining community-level resilience (Fig. 2). Material deprivation, constituting the third and most heavily weighted dimension (0.4), captures the extent to which financial and socioeconomic constraints prevent individuals or households from engaging in self-protective behaviours against asbestos-related risks. This includes the inability to afford housing remediation, health-related expenditures, or relocation from contaminated environments. The observed distribution again reveals stark contrasts across municipalities: C-1 reports the minimal score of 1.00, while C-5 registers the maximum of 5.00, with intermediate cases such as C-3 (2.22) and C-4 (2.80) reflecting a gradient of economic vulnerability.

**Fig. 3:**
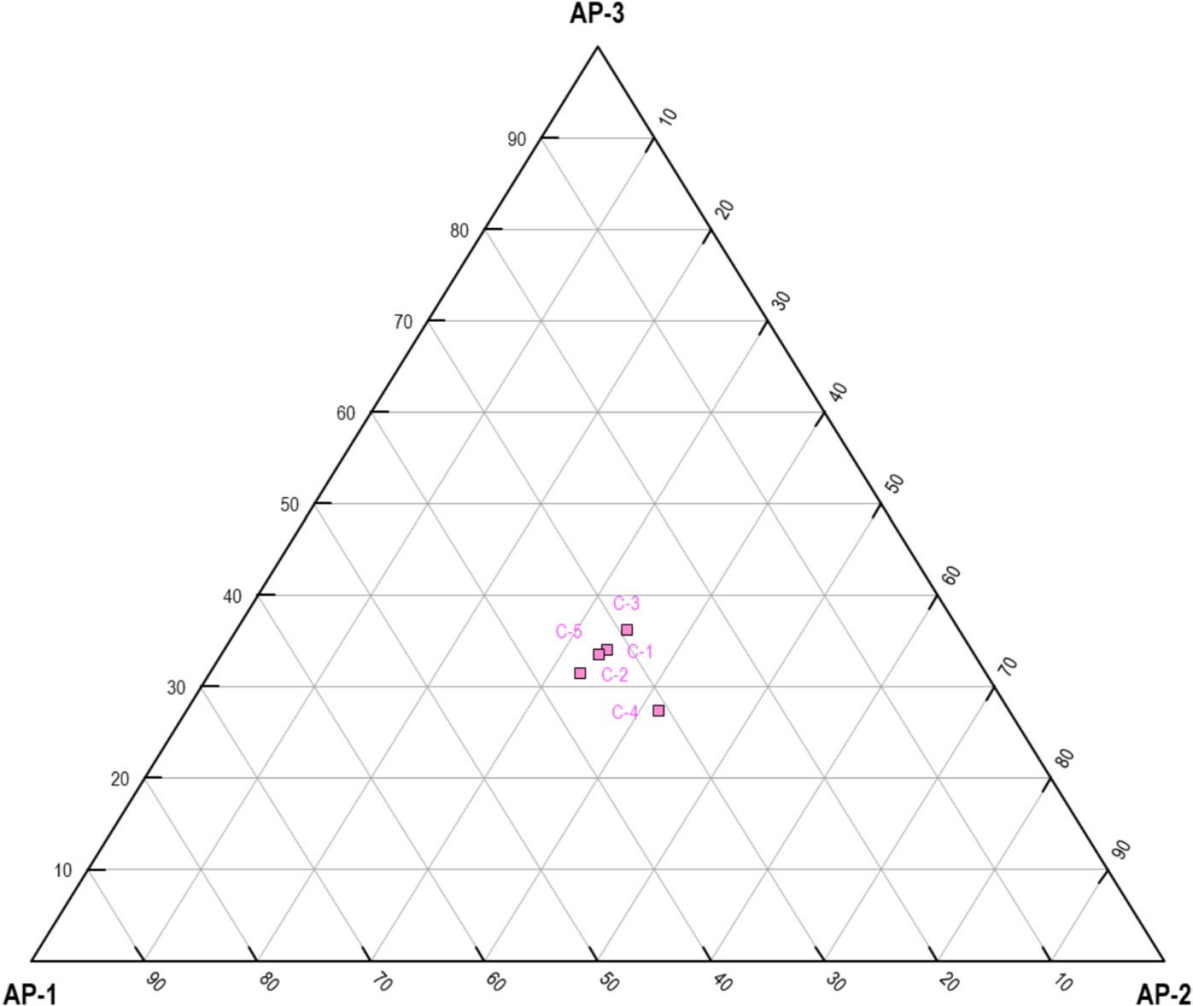
Focus areas and directions for urban planning based on AP values

Interestingly, in some cases (e.g. C-2, score: 3.40), material deprivation appears elevated even when service access is not the most limiting factor. This decoupling indicates that financial precarity alone can act as a bottleneck for risk mitigation, regardless of informational or infrastructural adequacy (Fig. 3). Given its amplified weight in the index, material deprivation exerts a decisive influence on the final AP score, often tipping the balance in municipalities with borderline values in the other two dimensions. This confirms the theoretical assumption that economic capital remains the most critical determinant of environmental resilience in the context of chronic exposure hazards.

## Discussion

Collectively, the multidimensional data support the hypothesis that AP is not reducible to a single determinant but emerges from the interaction of disparate forms of deprivation, each with unique spatial and functional logics. The structural independence of the three macro-areas is empirically evidenced by the asynchronous distribution of their values across municipalities. Furthermore, the composite index reveals non-linear accumulation effects, wherein simultaneous moderate deprivations in two or more domains may yield total index values comparable to those observed in municipalities with extreme scores in a single dimension. This has direct implications for intervention prioritization: targeted, domain-specific strategies are likely to be more effective than generalized approaches, especially in contexts where deprivation is driven by an identifiable structural deficit rather than uniform systemic failure. These findings also challenge the conventional reliance on income-based or service-based metrics alone when assessing environmental vulnerability.

In the context of AP, the informational dimension functions not merely as a background condition, but as a critical multiplier that modulates the efficacy of material and institutional capacities. As such, future public health planning must consider the dynamic interplay between these three degrees of deprivation to produce more equitable, evidence-based, and locality-sensitive interventions.

This research’s findings reveal substantial disparities in AP levels across the five examined municipalities, corroborating existing research on environmental justice and health inequities stemming from hazardous material exposure. By investigating three distinct dimensions—informational poverty, service accessibility, and material deprivation—the analysis identified significant variations in vulnerability, with some municipalities exhibiting effective mitigation approaches, while others remain acutely susceptible to asbestos-related risks. The variations in informational poverty across municipalities highlight the significance of effective public health communication strategies. Municipality C-1, with the lowest score, benefits from extensive outreach programmes and accessible asbestos-related knowledge. Analogous findings were reported by Etim [21], who emphasized that community engagement and transparent risk communication substantially mitigate health disparities. Conversely, the severe informational deprivation observed in C-5 aligns with previous studies demonstrating that marginalized communities often lack essential knowledge about environmental hazards, exacerbating their vulnerability [8, 54]. Moreover, studies underscore the role of misinformation and distrust in institutions as additional barriers to effective risk communication, further disadvantaging already vulnerable populations [69]. The second dimension of AP, service accessibility, highlighted critical gaps in healthcare and protective service availability, particularly in C-5. Extant research has established that access to healthcare services plays a pivotal role in mitigating asbestos-related illnesses, as early detection and intervention can significantly reduce morbidity and mortality [26, 61]. In contrast, the effective healthcare infrastructure in C-1, as evidenced by its low score, is consistent with previous studies demonstrating that well-funded public health systems correlate with lower rates of asbestos-related diseases. Conversely, municipalities with limited healthcare access, such as C-5, face heightened risk due to systemic barriers preventing timely medical intervention. Additionally, disparities in healthcare accessibility often coincide with geographic and infrastructural challenges, particularly in remote or underfunded regions, where specialized asbestos-related healthcare services are limited [84]. The third dimension of AP, material deprivation, demonstrates how financial limitations impede protective measures against asbestos exposure. Existing research indicates that economically disadvantaged populations face elevated exposure risks due to substandard housing and restricted access to protective resources [19, 27, 34, 50]. The severe material deprivation in C-5 suggests a direct correlation between poverty and asbestos vulnerability, echoing findings from prior studies on environmental health disparities. Conversely, the lower material deprivation score in C-1 implies fewer financial constraints, supporting the notion that economic stability enables risk mitigation efforts. Furthermore, the persistence of asbestos in lower-income housing stock underscores the need for targeted financial assistance programmes to support safe remediation efforts and housing improvements [42]. The final AP index underscores significant variations across the examined municipalities, with C-1 demonstrating the lowest risk profile and C-5 confronting the most severe challenges. These findings corroborate existing scholarship on socio-environmental vulnerability, highlighting the imperative for targeted policy interventions in high-risk areas [22, 53]. Given the strong association between AP levels and public health outcomes, policy frameworks should prioritize resource allocation to the municipalities exhibiting elevated AP scores. Fundamental strategies to mitigate these disparities include enhanced risk communication, improved healthcare accessibility, and economic support for impacted communities.

Ongoing research should investigate the long-term efficacy of intervention strategies in mitigating AP and associated health hazards. Longitudinal examinations of the impact of educational initiatives, policy modifications, and economic support mechanisms would offer valuable insights to inform policymaking. Moreover, incorporating community-engaged participatory research approaches can empower affected populations and ensure that interventions address local needs. Policy recommendations should emphasize cross-sectoral collaboration, involving public health agencies, environmental organizations, and municipal authorities, to develop comprehensive asbestos remediation strategies.

## Conclusions

These findings reveal pronounced disparities in AP across the five municipalities under investigation, reflecting deep-seated structural inequities in informational access, service availability, and material capacity to mitigate environmental health risks. The markedly lower AP index observed in municipality C-1 exemplifies the potential impact of cohesive public health systems, targeted risk communication, and supportive socio-political frameworks. In contrast, C-5 demonstrates acute multidimensional deprivation, illustrating how cumulative disadvantages can converge to exacerbate vulnerability to asbestos-related harm. This stratification in AP outcomes highlights the critical need for integrative, evidence-informed policy responses that recognize and address the layered nature of environmental health inequities.

Importantly, the multidimensional index applied in this paper underscores the necessity of addressing AP not merely as a consequence of economic scarcity but as a complex, intersecting phenomenon shaped by informational, institutional, and financial determinants. These dimensions are not only additive but often mutually reinforcing: limited awareness of asbestos hazards reduces engagement with risk mitigation programmes, while gaps in service provision and institutional support can perpetuate cycles of exclusion and exposure. In this regard, policy interventions must move beyond sectoral approaches and instead adopt systemic, cross-disciplinary strategies that reflect the socioecological embeddedness of environmental health outcomes.

To meaningfully reduce AP, municipalities, particularly those registering higher index scores, must pursue targeted interventions that enhance the accessibility and efficacy of both informational and operational infrastructures. This includes scaling up localized public education campaigns on asbestos risks, expanding the geographic and economic reach of remediation services, and redesigning financial assistance mechanisms to lower the threshold for programme participation among low-income households. Policy frameworks should also incorporate context-sensitive indicators capable of capturing localized risk dynamics and infrastructural deficits, thereby enabling more granular and responsive planning.

Furthermore, sustained intersectoral collaboration is essential. Urban planning must be informed by epidemiological risk profiling, public health institutions should coordinate with waste management and environmental safety agencies; and civil society actors must be empowered to play an active role in risk communication and community mobilization. The integration of geospatial analytics, health impact assessments, and participatory governance mechanisms could also prove pivotal in designing interventions that are not only effective but also equitable and socially legitimate. While the presented results provide compelling evidence for the urgent need to address AP through multidimensional, locally tailored strategies, it is equally crucial to consider the practical applicability and current limitations of this research. A key strength of the proposed framework lies in its operational versatility: the AP index developed here is designed to function both as a diagnostic tool and a policy support mechanism. It allows municipalities to identify key dimensions of deprivation, whether informational, infrastructural, or material, and to prioritize interventions based on localized risk configurations. In this sense, the index offers a scalable, adaptable approach that can be applied across varied urban contexts and governance structures. Nonetheless, the findings also point to a major systemic challenge: a widespread lack of institutional awareness and preparedness regarding asbestos-related risks. Currently, only a limited number of municipalities acknowledge the issue in strategic terms, and even fewer possess coherent, evidence-based frameworks to assess and mitigate AP. This institutional invisibility of asbestos risks is not merely a reflection of limited resources but also of insufficient regulatory incentives and fragmented knowledge ecosystems. Such gaps are mirrored in the constrained sample size of the present paper, which was necessarily limited to five municipalities where preliminary awareness or data availability allowed for a meaningful application of the proposed methodology. While this limited sample size restricts the generalizability of specific index values, it also fulfils a critical methodological function: it provides a proof of concept for a novel evaluative framework capable of integrating diverse dimensions of socio-environmental vulnerability. The empirical application thus serves as an initial foundation upon which more comprehensive, large-scale methodologies may be developed. If adopted and further refined, the AP index can contribute to the standardization of asbestos risk assessment across jurisdictions and foster inter-municipal comparability in future research.

Moreover, this work holds the potential to stimulate scholarly interest and interdisciplinary dialogue on the broader implications of environmental health deprivation. By foregrounding the need for cross-cutting indicators and integrated risk governance, the paper positions itself as a catalyst for further methodological innovation in the field. Encouragingly, the structured articulation of informational, infrastructural, and material domains aligns with growing international efforts to develop holistic environmental justice metrics, efforts to which this research aims to contribute. For these reasons, further validation and expansion of the AP index are warranted. Future applications should aim to include a more diverse range of municipalities, integrate longitudinal data to assess dynamic risk trajectories, and incorporate qualitative insights from affected communities. In parallel, partnerships with national health and environmental agencies could help institutionalize the framework and enhance its practical relevance for local-level decision-makers. As such, this paper not only addresses a critical gap in current asbestos governance but also offers a scalable platform for future research and policy development, provided that it captures the attention and collaboration of the scientific and policy communities alike. Given the differentiated nature of AP across the studied municipalities, future research should investigate the long-term outcomes of existing mitigation programmes and assess the replicability of best practices observed in lower-index areas. In parallel, cost-effectiveness analyses of proposed intervention models could provide policymakers with critical data for resource allocation. Moreover, attention must be paid to the evolving regulatory context, particularly in relation to European Union directives on hazardous material management, which may influence both the strategic direction and operational feasibility of municipal asbestos remediation efforts. Ultimately, the reduction of AP requires a proactive and anticipatory policy ethos, one that anticipates environmental justice implications and centres equity as a core evaluative criterion. By embracing this holistic approach, municipalities can transition from reactive crisis management to the sustained protection of public health, thereby fostering more resilient and just urban environments. The evidence presented in this paper affirms the urgency of such a shift and provides a conceptual and empirical foundation for its realization.

## Data Availability

The datasets generated during and analyzed during the current study are not publicly available but are available from the corresponding author on reasonable request.
